# A clinical case study of the use of ecological momentary assessment in obsessive compulsive disorder

**DOI:** 10.3389/fpsyg.2014.00339

**Published:** 2014-04-17

**Authors:** P. J. Matt Tilley, Clare S. Rees

**Affiliations:** Brain, Behaviour and Mental Health Research Group, School of Psychology and Speech Pathology, Curtin UniversityPerth, WA, Australia

**Keywords:** obsessive-compulsive disorder (OCD), ecological momentary assessment, ecological momentary assessment data, anxiety disorders, assessment

## Abstract

Accurate assessment of obsessions and compulsions is a crucial step in treatment planning for Obsessive-Compulsive Disorder (OCD). In this clinical case study, we sought to determine if the use of Ecological Momentary Assessment (EMA) could provide additional symptom information beyond that captured during standard assessment of OCD. We studied three adults diagnosed with OCD and compared the number and types of obsessions and compulsions captured using the Yale-Brown Obsessive-Compulsive Scale (Y-BOCS) compared to EMA. Following completion of the Y-BOCS interview, participants then recorded their OCD symptoms into a digital voice recorder across a 12-h period in reply to randomly sent mobile phone SMS prompts. The EMA approach yielded a lower number of symptoms of obsessions and compulsions than the Y-BOCS but produced additional types of obsessions and compulsions not previously identified by the Y-BOCS. We conclude that the EMA-OCD procedure may represent a worthy addition to the suite of assessment tools used when working with clients who have OCD. Further research with larger samples is required to strengthen this conclusion.

## Introduction

Obsessive-Compulsive Disorder (OCD) is a disabling anxiety disorder characterized by upsetting, unwanted cognitions (obsessions) and intense and time consuming recurrent compulsions (American Psychiatric Association, 2000). The idiosyncratic nature of the symptoms of OCD (Whittal et al., 2010) represents a challenge to completing accurate and comprehensive assessments, which if not achieved, can have a deleterious effect on the provision of effective treatment for the disorder (Kim et al., 1989; Taylor, 1995; Steketee and Barlow, 2002; Deacon and Abramowitz, 2005).

Accurately assessing the full range of symptoms of OCD requires reliable and psychometrically sound diagnostic instruments and measures (Taylor, 1995, 1998; Rees, 2009) alongside the standard clinical interview. Although the most commonly used psychometric instrument for assessing OCD, the Yale-Brown Obsessive Compulsive Scale (Y-BOCS) (Goodman et al., 1989a,b), has acceptable reliability and convergent validity, it has been criticized by Taylor (1995) for weak discriminant validity. Taylor also highlighted that it remains susceptible to administration variance, relies on client memory recall, and is time consuming to administer. As with all measures completed retrospectively, selective memory biases affect the type of information reported by clients about their symptoms (Clark, 1988; Stone and Shiffman, 2002; Stone et al., 2004). Glass and Arnkoff (1997, p. 912) have summarized several disadvantages of structured inventories; first, they contain prototypical statements which may fail to capture the idiosyncratic nature of the client's actual thoughts; second, they can be affected by *post-hoc* reappraisals of what clients feel, as the data is subject to memory recall biases; and finally they may fail to adequately capture the client's internal dialog due to the limitations of the best fit question structure.

Discrepancies have been reported between data collected in the client's natural environment (*in situ*) and those based on the client's later recall (de Beurs et al., 1992; Marks and Hemsley, 1999; Stone et al., 2004). Such discrepancies may be further affected by factors such as the complexity and diversity of obsessions and compulsions, not to mention the ego-dystonic nature of many OCD clients' obsessional thoughts. It seems likely that clients with distressing ego-dystonic obsessions, for example, those involving sexual, aggressive, and/or religious themes may experience a heightened level of discomfort in reporting their obsessions in a face to face assessment with a clinician, thus reducing their willingness to accurately report (Taylor, 1995; Newth and Rachman, 2001; Grant et al., 2006; Rees, 2009). This may contribute to an underreporting of these obsessions, and hence an inaccurate understanding and a restriction of the clinician's ability to adequately treat the client (Grant et al., 2006; Rachman, 2007).

Exposure and response prevention, cognitive therapy, and pharmacological interventions have been shown to be effective in the treatment of OCD (Abramowitz, 1997, 2001; Foa and Franklin, 2001; Steketee and Barlow, 2002; Fisher and Wells, 2008; Chosak et al., 2009). Self-monitoring is a useful therapeutic technique that provides essential information to assist in the development of exposure hierarchies and behavioral experiments used in cognitive therapy (Tolin, 2009). Clients typically observe and record their experiences of target behaviors, including triggers, environmental events surrounding those experiences, and their response to those experiences (Cormier and Nurius, 2003). Such self-monitoring can be used to both assist assessment and/or as an intervention. Cormier and Nurius (2003) explained that the mere act of observing and monitoring one's own behavior and experiences can produce change. As people observe themselves and collect data about what they observe, their behavior may be influenced.

A form of self-monitoring and alternative to the typical clinic-based assessment of OCD is the use of sampling from the client's real-world experiences, a procedure known as Ecological Momentary Assessment (EMA) (Schwartz and Stone, 1998; Stone and Shiffman, 1994, 2002). EMA does not rely on measurements using memory recall within the clinical setting, but rather allows for collection of information about the client's experiences in their natural setting, potentially improving the assessment's ecological validity (Stone and Shiffman, 2002). *In situ* sampling techniques have been successfully used in psychology, psychiatry, and occupational therapy (for a more detailed account see research by Morgan et al., 1990; de Beurs et al., 1992; Kamarack et al., 1998; Litt et al., 1998; Kimhy et al., 2006; Gloster et al., 2008; Putnam and McSweeney, 2008; Trull et al., 2008). Generally it is agreed that EMA offers broader assessment within the client's natural environment, as it includes random time sampling of the client's experience, recording of events associated with the client's experience, and self-reports regarding the client's behaviors and physiological experiences (Stone and Shiffman, 2002). Because this assessment method accesses information about the client's situation, the difficulties of memory distortions like recall bias are reduced (Schwartz and Stone, 1998; Stone and Shiffman, 2002).

Given that accurate assessment of obsessions and compulsions is a critical aspect of treatment planning and that reliance on self-report and clinician interview has some known limitations, the purpose of this study was to investigate the utility of EMA as a potential adjunct to the conventional assessment of OCD. Specifically, we sought to compare the amount and type of information regarding obsessions and compulsions collected via EMA vs. standard assessment using the gold-standard symptom interview for OCD. As this is a pilot clinical case study, we offer the following tentative hypothesis: (1) EMA will yield additional types of obsessions and compulsions not captured by the Y-BOCS.

## Background

### Participants and setting

Participants were recruited through clients presenting to the OCD clinic at Curtin University. They were assessed using the Structured Clinical Interview for DSM-IV (SCID-IV) (First et al., 1997). Inclusion in the study was based on receiving a primary diagnosis of OCD, and a Y-BOCS (Goodman et al., 1989a,b) score of more than 16, placing their OCD symptom severity within the clinical range (Steketee and Barlow, 2002). Participants were excluded if they presented with current suicidal ideation, psychotic disorders, apparent organic causes of anxiety, were severely depressed, or if they had an intellectual disability. One potential participant was excluded post evaluation despite meeting the inclusion criteria, as she did not own a mobile phone, and reported having “blackouts” throughout the day. The three participants all had OCD symptoms in the “severe” range according to the YBOCS. In order to ensure that participants remain anonymous, pseudonyms have been used.

#### Participant A

Mary was a 28-year-old female who lived with her husband and small dog. She reported that for approximately 1 year she had been experiencing distressing intrusive thoughts in relation to harming her loved ones, herself, or her dog; for example, by stabbing, electrocution, or breaking the dog's neck. Mary said that she also had reoccurring thoughts and images that her husband or other family members might die. She reported engaging in some rituals, for example straightening pillows and rearranging tea-towels; but mostly reported using “safety nets” in response to her unwanted cognitions; for example ensuring that she was not alone (to prevent self-harm); avoidance and removal of feared object; extensive reassurance seeking from family members. According to the Y-BOCS measure, Mary scored a subtotal of 14 for Obsessions and a subtotal of 18 for Compulsions, giving an overall total of 32, classifying her symptoms as “severe” (Steketee and Barlow, 2002).

Mary reported that her OCD first occurred after her grandmother passed away about 6 years ago. She explained they had a very close relationship, she said she found it “unbearably distressing” to visit her while she was dying. Mary reported that on one occasion whilst in a coma, her grandmother sat up and gasped, which she found extremely frightening and still remembers it in vivid detail. She reported that she experienced thoughts that her grandmother was in pain and was going “into the unknown, to a scary place.” Mary reported feeling afraid of death and that if someone “even closer” to her died she “would not be able to cope” and that she would “lose control completely.” She stated that her biggest fear was that her husband, mother or father might die. Mary reported that she has been on various anti-depressants for about 10 years. She stated that recently her psychiatrist prescribed Solian (an antipsychotic) which she tried, and found was very effective at blocking out the intrusive thoughts. However, she ceased taking the medication due to nausea.

#### Participant B

John was a 5-year-old man who lived with his wife and adult son. He reported a long history of distressing intrusive thoughts, and compulsive behaviors. They are summarized in three ways. First, those that relate to religious obsessions, specifically the occult and satanic experiences/fear of being “possessed.” He reported responding to these unwanted cognitions by either washing his hands to cleanse himself; using more than six pieces of toilet paper to wipe after defecating to prevent the devil entering him via his anus; or looking for the number “555,” which represents “God. This is good.” John reported that failing to act in these ways would risk causing harm to his wife and son. Second, those that relate to checking compulsions, specifically when driving, and also checking that doors are locked—which he reported doing 4–12 times per night. He reported that if he thought he heard a “bump” when driving he would have to turn back to check he had not run anyone over, or would seek reassurance from his son or wife if they were passengers in the car with him. He stated that he feared that harm would come to his wife and son if he didn't perform these checks. Third, John said that he arranged shoes so that they were “lined up” and that the clothes in the cupboard were in the “right order.” He also reported the need to compulsively clean his son's bedroom, and that he wouldn't feel “right” until he had done so. According to the Y-BOCS measure, John scored a subtotal of 13 for Obsessions and a subtotal of 15 for Compulsions, giving an overall total of 28, classifying his symptoms as “severe” (Steketee and Barlow, 2002).

John reported that his symptoms have been present for at least the last 29 years. He reported that his OCD first occurred after he had a “break-down” and tried to commit suicide by stabbing himself in the stomach before he turned 25 years of age. In the years leading up to this, John reported two poignant experiences which appear relevant to the development of his symptoms; he reported being involved in the euthanizing of two dogs whilst working as a Ranger's assistant; and that when he was young, he and his girlfriend at the time had a pregnancy termination. John reported feeling that these were “blasphemous” acts, and posed the question “Is God punishing me?” John reported that he had been on several different anti-depressants for about 19 years, with varying degrees of success and side-effects. He reported that he had seen a psychiatrist every 6 weeks for “many years” and finds being able to talk helpful.

#### Participant C

Paul was a 35-year-old man, who reported distressing intrusive thoughts and images in relation to harm coming to others as a consequence of him not checking that he had done what he is “supposed to do.” For example, he was concerned that someone at work would be harmed if he forgot to adequately cover shifts on the roster (something he is responsible for); or when a client of the service he coordinates was recently given a stereo, Paul reported that he feared that harm would come to the client if he didn't correctly check it to see if it was faulty, something he felt responsible to do.

Paul reported that only his partner knew of his difficulties. He stated that he did not allow his anxiety to interfere too much with his occupational functioning; however he did report that the main reason he does not practice in his profession is because of his OCD. According to the Y-BOCS measure, Paul scored a subtotal of 12 for Obsessions and a subtotal of 13 for Compulsions, giving an overall total of 25, classifying his symptoms as “severe” (Steketee and Barlow, 2002).

Paul reported that his intrusive thoughts have “always been there.” He explained that one of the first clear memories he has of them, was when he was seven years old and he saw the film the “The Omen.” He reported remembering checking his head for the numbers “666.” Additionally, he reported remembering that he was concerned for his mother's safety. He reported that he had never taken medication for his OCD. He stated that he saw two therapists when he lived in the UK at an OCD center in London approximately 18 months ago. Paul said that he did not gain much from the first therapist, but believes that second therapist assisted him to look at his cognitions as “just thoughts.”

## Materials and methods

All screening of participants, interviewing and assessment, as well as administration of the study, was conducted by the first author, who was a provisionally registered psychologist undergoing postgraduate training at the time of the research, and was supervised by the second author, an experienced OCD clinician and academic. Potential participants were recruited from the Curtin OCD clinic. They were screened via telephone to ascertain their suitability for the study. A face-to-face assessment session using the Structured Clinical Interview for DSM-IV (SCID-IV; First et al., 1997) was conducted to determine a primary OCD diagnosis, followed by the administration of the Yale-Brown Obsessive-Compulsive Scale (Y-BOCS) interview and checklist (Goodman et al., 1989b) to identify the participants' obsessions and compulsions and their symptom severity. The Y-BOCS is the most widely used scale for OCD symptoms assessment and is considered by researchers to be the “gold standard” measure for symptom severity (Deacon and Abramowitz, 2005; Himle and Franklin, 2009). It consists of two parts; a checklist of prelisted types of obsessions, usually endorsed by the clinician based on disclosures made by the client; and the severity scale which requires the client to rate the severity of their experience by answering the questions based on their recall. Goodman et al. (1989) note that the Y-BOCS has shown adequate interrater agreement, internal consistency, and validity.

Suitable participants then attended a second session where they signed consent forms and were given instructions about the study procedure. During the data collection using the Ecological Momentary Assessment data (EMA-OCD), participants used an Olympus WS-110 digital voice recorder to record their experiences throughout a 12 h period. Participants used their existing mobile phones to receive prompts via the mobile phone Short Message Service (SMS) to record their responses to the research questions. All three participants were then provided with an envelope containing the Olympus WS-100 digital voice recorder, a spare battery, and the participant prompt questions (see Table 1).

**Table 1:** **Prompt questions**.

1. Since the last prompt have you experienced any unwanted thoughts?
2. If YES, please describe it/them to the recorder
• What form did it/they take e.g. an image, a thought, a word, etc
• Did something trigger it/them?
3. Since the last prompt have you felt compelled to do anything in response to your unwanted thoughts, either physically, verbally, or mentally?
4. If YES, please describe it/them to the recorder

Participants were asked to turn their mobile phones on during the data collection day by 10 am, ready to receive their SMS prompts. The researcher manually sent SMS prompts to the participants at random intervals; at least every 2 h (across 1 day, from 10 am to 10 pm), for a minimum of 10 data entries in keeping with research using EMA procedures (see, Stone and Shiffman, 2002); asking them to complete their responses to all four questions as details on the EMA-OCD Participant Questions Sheet. Participants were instructed not to respond to the SMS prompts if driving, and were asked to respond as quickly as possible to the prompts. Data was then downloaded from the voice recorder to the researcher's computer, and transcribed. During this process all identifying details were removed. During the debrief session open-ended questions were used to gather as much information as possible regarding the participant's experiences of the study, and suggestions for improvements. During the data collection day the researcher completed a journal to record his observations and reflections related to the use of the EMA. At the completion of the EMD-OCD data collection, each of the participants was provided with a debrief session (Mary by phone, and John and Paul, face to face). The debrief session focused on their experiences of the research and use of the digital voice recorder; and provided the opportunity for them to discuss anything else that arose they wished to tell the researcher. As stated above, the data was downloaded and transcribed by the first author. The Y-BOCS obsession and compulsion categories were used as a framework to compare the data generated from the EMA-OCD procedure. After the complete de-identified data set was tabled, it was provided to a second person who was an expert in OCD for verification of categories. In the case of any discrepancies agreement was reached via consensus.

### Findings

#### Number of reported symptoms

Table 2 provides a summary of the frequency and type of symptoms recorded during both the face-to-face session, which will be referred to as the Y-BOCS data and the EMA-OCD phase for the study, which will be referred to as the EMA–OCD data. As can be seen when comparing the data contained in the two columns, there are variations between the Y-BOCS data and the EMA-OCD data. All three participants reported more categories of both obsessions and compulsions in the Y-BOCS data, compared to that reported in the EMA-OCD data.

**Table 2:** **Summary by participant of Y-BOCS data and EMA-OCD data**.

	**Y-BOCS Data**	**EMA-OCD Data**
**PARTICIPANT A—MARY**		
**Obsessions/Intrusive thoughts**		
Aggressive obsessions		
• Fear might harm self		
• Stab herself	✓	✓
• Swallow the contents of a bottle of lavender^*^		✓
• Fear might harm others		
• Kill the dog	✓	✓
• Fear that husband might die as a result of not completing compulsions	✓	✓
• Fear that husband will die if she doesn't wait for the microwave to complete it's cycle to the minute^*^		✓
• Fear of blurting out obscenities	✓	
• Fear will act on unwanted impulses	✓	
Contamination obsessions		
• Other – Fear that food will be contaminated e.g. lettuce	✓	
Hoarding/Saving obsessions		
• Saving unwanted gifts for the house	✓	
Obsession with need for Symmetry or Exactness		
• Fears “bad things” will happen if shoes, ruler, biros are not lined up and in the “right” place	✓	
• Fears “bad things” will happen if tea-towel or cloths are not arranged “correctly”	✓	✓
• Fears “someone will die” if she didn't drive as close to the bin as possible^*^		✓
Miscellaneous obsessions		
• Fear of losing things	✓	
• Lucky/Unlucky numbers	✓	
• Colours with special significance / Superstitious fears	✓	
**Compulsions/Rituals/Responses**		
Checking compulsions		
• Checking locks, stoves, appliances, etc	✓	
• Checking that did not make a mistake	✓	
Repeating rituals		
• Re-reading or re-writing	✓	
• Need to repeat routine activities e.g. shutting the door several times; & arranging pillows on the couch	✓	
Ordering/Arranging compulsions		
• Need to arrange household items e.g. shoes, ruler, biro	✓	✓
• Need to fold toilet paper “correctly”^*^		✓
Miscellaneous compulsions		
• Need to tell, ask, or confess	✓	
• Ritualised eating behavior	✓	
Avoidance		
• Distraction	✓	✓
• Keeping busy	✓	✓
• Over sleeping	✓	
Reassurance seeking		
• From husband and family members	✓	
Thought suppression		
• “Just tried to get the thought out of my head”^*^		✓
**PARTICIPANT B—JOHN**		
**Obsessions/Intrusive thoughts**		
Aggressive obsessions		
• Fear will be responsible for something terrible happening	✓	
Religious obsessions		
• Fear of being possessed by the devil, or satanic forces	✓	✓
Obsession with need for Symmetry or Exactness		
• Shoes need to be lined up and in the right place – states that there is no fear of bad things happening, but it “just wouldn't feel right”	✓	✓
• Clothes need to be hung “correctly” – otherwise again it “just wouldn't feel right”	✓	✓
Miscellaneous obsessions		
• Fear of not saying just the right thing	✓	
• Lucky/Unlucky numbers	✓	
• Colors with special significance/Superstitious fears	✓	
**Compulsions/Rituals/Responses**		
Cleaning / Washing compulsions		
• Excessive or ritualised hand-washing	✓	
• Excessive or ritualised house cleaning	✓	
• Excessive or ritualised wiping after defecating	✓	✓
• Excessive or ritualised showering^*^		✓
Checking compulsions		
• Checking locks, stoves, appliances, etc	✓	
• Checking that did not/will not harm others	✓	
Counting compulsion		
• Reported turning his C-PAT machine on and off 7 times to avoid a derivative of 6^*^		✓
Ordering/Arranging compulsions		
• Need to arrange household items “in just the right order” e.g. shoes need to be lined up; clothes to be hung “correctly”	✓	
• Need to make house clean and tidy, with special focus on his son's bedroom	✓	
Miscellaneous compulsions		
• Need to touch the crucifix to cleanse himself^*^		✓
Avoidance		
• Distraction	✓	✓
• Keeping busy	✓	
Reassurance seeking		
• From wife and son	✓	
Thought suppression		
• “Numbing them (the IT) out of my head”^*^		✓
• “Dismissing them (the IT)”^*^		✓
**PARTICIPANT C— PAUL**		
**Obsessions/Intrusive thoughts**		
Aggressive obsessions		
• Fear of doing something else embarrassing	✓	✓
• Fear will act on unwanted impulses	✓	✓
• Fear will harm others because not careful enough	✓	✓
• Fear will be responsible for something terrible happening	✓	✓
• Fear he will be accused of stealing something^*^		✓
Sexual obsessions		
• Forbidden or perverse sexual thoughts, images, impulses	✓	
• Content involves children or incest	✓	
Religious obsessions		
• Excessive concern with right/wrong, morality	✓	
Miscellaneous obsessions		
• Fear of losing things	✓	
• Intrusive (non-violent) images—all sexual	✓	
**Compulsions/Rituals/Responses**		
Checking compulsions		
• Checking locks, stoves, appliances, etc	✓	✓
• Checking that did not/will not harm others	✓	✓
• Checking that nothing terrible did/will happen	✓	✓
• Checking that did not make a mistake	✓	✓
Repeating rituals		
• Re-reading or re-writing	✓	
Miscellaneous compulsion		
• Playing a CD in the “correct” order^*^		✓

* *, Represents a new category of either Obsessions or Compulsions*.

Mary reported experiencing five categories of Y-BOCS Obsessions and six categories of Compulsions in the Y-BOCS data. In the EMA-OCD data she reported experiencing two categories of Y-BOCS Obsessions, and three categories of Y-BOCS Compulsions. John reported experiencing four categories of Y-BOCS Obsessions and five categories of Compulsions in the Y-BOCS data. In the EMA-OCD data he reported experiencing two categories of Y-BOCS Obsessions, and five categories of Y-BOCS Compulsions. Paul reported experiencing four categories of Y-BOCS Obsessions and two categories of Compulsions in the Y-BOCS data. In the EMA-OCD data he reported experiencing one category of Y-BOCS Obsessions, and two categories of Compulsions.

#### Comparison of content of symptoms

Both Mary and Paul reported previously unidentified Obsessions or intrusive thoughts in the EMA-OCD data, compared to the Y-BOCS data; and all three participants reported previously unidentified compulsions/rituals/responses in the EMA-OCD data. As can be seen in Table 2, Mary reported two intrusive thoughts in the EMA-OCD data that were not recorded in the Y-BOCS data. Additionally, she reported a previously unreported obsession under the obsession category *Obsession with need for Symmetry or Exactness*, not reported in the Y-BOCS data. Mary also reported variations on her compulsive behaviors and the presence of thought suppression not identified during the administration of the Y-BOCS. The EMA-OCD data indicated that John substituted one of his compulsions for an alternative anxiety reducing act, which was not recorded in the Y-BOCS data and suggests the identification of a previously unreported compulsion. Additionally, the EMA-OCD data indicated that John engaged in thought suppression to neutralize his intrusive thoughts. Likewise, John's reported compulsive behaviors also varied between data sets. In the EMA-OCD data he reported three previously unreported compulsive behaviors, and like Mary also the presence of thought suppression. In addition to the above, the EMA-OCD data indicated that John substituted one of his rituals for another, when he touched a crucifix instead of performing his usual hand washing ritual to cleanse him-self of the potential satanic possession. This was not something reported in the Y-BOCS data.

## Discussion

This study investigated the utility of EMA as an adjunct assessment approach for OCD. Each of our study hypotheses was supported. As predicted the EMA procedure resulted in the identification of additional types of obsessions and compulsions not captured by the Y-BOCS interview. The finding that the EMA procedure identifies obsession and compulsion symptoms not captured by the Y-BOCS suggests that further studies in this area are warranted. As a pilot case study we cannot generalize from these initial findings but our results indicate that a larger study replicating the procedure used here, is justified. Importantly, the three participants in our study were representative of quite typical OCD clients in that they had severe levels of symptoms and had OCD for a number of years. The EMA procedure we used was found to be satisfactory to all three participants. Feedback from the participants at the de-briefing session included suggestions that this process would be helpful for therapy because it would provide the therapist and client with rich and current material regarding their symptom patterns. From a clinician's point of view, collecting the EMA data is not onerous because the entries are simply short answers collected on 12 occasions and thus is not a time-consuming exercise.

The EMA procedure as used in this study could provide clinicians with a new method by which to gain a current and accurate snap-shot of clients symptoms as they occur in real-time. This information could augment information gained from standard pencil and paper measures but also provide an “active” process which may help to engage clients in the therapeutic process. It seems likely that using a procedure like EMA with OCD clients will assist in understanding their OCD experiences, and thus assist in generating valuable information, supporting accurate assessment, client conceptualization, and ultimately treatment.

Despite these valuable findings, there are limitations of this study. As a pilot study and exploratory in nature, it is only possible to draw limited interpretations from the data provided. However, the preliminary findings of this study support the benefit of conducting further research into this procedure, where it may be possible to draw more empirically valid findings from a larger and more statistically powerful sample. Second, due to the lack of availability of date stamping, participants were asked to record the time they made each recording. Unfortunately this was not routinely provided by all participants, and hence creates an unanswerable question regarding the accuracy of the data recorded. As Stone and Shiffman (2002) discuss, a potential problem relates to participants recording their data based on their recall of what was occurring at the time of the SMS prompt, rather than immediately. Hence introducing possible memory bias, and undermining the premise of the study. Although this is certainly an unwanted variable, based on the EMA-OCD data provided it seems that except for Mary, both John and Paul responded promptly to the SMS messages, or recorded the time if they didn't. Mary on the other hand, reported during the debrief session that she was unable to record the time for the initial targets, but did so for subsequent SMS prompts. It was not possible to ascertain from her data the delay in time between the first SMS prompts and her recordings. In future applications of this procedure, it is recommended that the device used provides automatic date-stamping to address this limitation. Indeed, it may be possible to adapt the EMA methodology for use with smart phones via a dedicated OCD application.

## Concluding remarks

The findings from this study of three patients with severe OCD suggest that the use of EMA provides important additional information regarding obsessions and compulsions and may thus be a useful adjunct to the clinical assessment of OCD.

### Conflict of interest statement

The authors declare that the research was conducted in the absence of any commercial or financial relationships that could be construed as a potential conflict of interest.
